# Supplementary material for: Multi-View Variational Autoencoder for Missing Value Imputation in Untargeted Metabolomics
Source: ArXiv. 2023 Oct 12:arXiv:2310.07990v1. Preprint. [Version 1] (PMC10593076)
Supplement: Supplementary file 1 [file NIHPP2310.07990V1-supplement-1.pdf]

Supplementary materials.

Table S1. Performance of metabolomics imputation using different number of templates by the proposed MVAE. The count of imputed metabolites achieved R^2-scores greater than thresholds are presented.

k	5	10	15	20	25	30	35	40	45	50
0.01	219	261	272	323	334	339	332	343	332	338
0.05	81	149	165	253	241	259	260	261	264	272
0.1	19	86	89	180	169	193	204	195	197	211
0.15	15	63	67	139	126	158	164	156	157	175
0.2	11	44	42	104	94	132	125	131	120	140

Table S2. Performance of metabolomics imputation using different number of templates by the proposed MVAE. The percent of imputed metabolites achieved R^2-scores greater than thresholds are presented.

k	5	10	15	20	25	30	35	40	45	50
0.01	44.51	54.26	56.31	71.46	70.32	72.13	71.55	74.08	72.33	74.29
0.05	16.46	30.98	34.16	55.97	50.74	55.11	56.03	56.37	57.52	59.78
0.1	3.86	17.88	18.43	39.82	35.58	41.06	43.97	42.12	42.92	46.37
0.15	3.05	13.10	13.87	30.75	26.53	33.62	35.34	33.69	34.20	38.46
0.2	2.24	9.15	8.70	23.01	19.79	28.09	26.94	28.29	26.14	30.77

Table S3. Performance of metabolomics imputation using different number of templates by the proposed MVAE. The count of imputed metabolites achieved MAPEs smaller than thresholds are presented.

k	5	10	15	20	25	30	35	40	45	50
0.1	1	1	2	1	2	1	2	1	1	2
0.15	12	17	20	18	20	21	22	21	19	20
0.2	40	47	50	49	54	51	52	47	49	52
0.3	111	118	120	124	134	134	135	130	131	130

Table S4. Performance of metabolomics imputation using different number of templates by the proposed MVAE. The percent of imputed metabolites achieved MAPEs smaller than thresholds are presented.

k	5	10	15	20	25	30	35	40	45	50
0.1	0.20	0.21	0.41	0.22	0.42	0.21	0.43	0.22	0.22	0.44
0.15	2.44	3.53	4.14	3.98	4.21	4.47	4.74	4.54	4.14	4.40
0.2	8.13	9.77	10.35	10.84	11.37	10.85	11.21	10.15	10.68	11.43
0.3	22.56	24.53	24.84	27.43	28.21	28.51	29.09	28.08	28.54	28.57

Table S5. Performance of metabolomics imputation using different methods with 35 template metabolites. The count of imputed metabolites achieved R^2-scores greater than thresholds are presented.

method	MVAE	KNN	Ridge	SVM	RF	GBR	MCCA	KCCA	KGCCA
0.01	332	218	326	251	226	316	280	260	253
0.05	260	183	255	194	181	220	220	196	194
0.1	204	145	202	151	148	166	166	150	137
0.15	164	121	152	124	124	136	127	119	107
0.2	125	100	122	98	94	107	114	97	78

Table S6. Performance of metabolomics imputation using different methods with 35 template metabolites. The percent of imputed metabolites achieved R^2-scores greater than thresholds are presented.

method	MVAE	KNN	Ridge	SVM	RF	GBR	MCCA	KCCA	KGCCA
0.01	71.55	46.98	70.26	54.09	48.71	68.10	60.22	56.03	54.41
0.05	56.03	39.44	54.96	41.81	39.01	47.41	47.31	42.24	41.72
0.1	43.97	31.25	43.53	32.54	31.90	35.78	35.70	32.33	29.46
0.15	35.34	26.08	32.76	26.72	26.72	29.31	27.31	25.65	23.01
0.2	26.94	21.55	26.29	21.12	20.26	23.06	24.52	20.91	16.77

Table S7. Performance of metabolomics imputation using different methods with 35 template metabolites. The count of imputed metabolites achieved MAPE smaller than thresholds are presented.

method	MVAE	KNN	Ridge	SVM	RF	GBR	MCCA	KCCA	KGCCA
0.1	2	0	1	0	0	1	1	1	1
0.15	22	13	21	16	12	20	19	16	15
0.2	52	45	50	45	44	47	47	44	45
0.3	135	122	131	116	112	126	126	120	116

Table S8. Performance of metabolomics imputation using different methods with 35 template metabolites. The percent of imputed metabolites achieved MAPE smaller than thresholds are presented.

method	MVAE	KNN	Ridge	SVM	RF	GBR	MCCA	KCCA	KGCCA
0.1	0.43	0.00	0.22	0.00	0.00	0.22	0.22	0.22	0.22
0.15	4.74	2.80	4.53	3.45	2.59	4.31	4.09	3.45	3.23
0.2	11.21	9.70	10.78	9.70	9.48	10.13	10.11	9.48	9.68
0.3	29.09	26.29	28.23	25.00	24.14	27.16	27.10	25.86	24.95
